# Sensitivity to Geometric Shape Regularity Emerges Independently of Vision

**DOI:** 10.1162/OPMI.a.39

**Published:** 2025-10-17

**Authors:** Andrea Adriano, Mathias Sablé-Meyer, Lorenzo Ciccione, Minye Zhan, Stanislas Dehaene

**Affiliations:** Cognitive Neuroimaging Unit, CEA, INSERM, Université Paris-Saclay, NeuroSpin center, Gif/Yvette, France; Collège de France, Université Paris Sciences Lettres (PSL), Paris, France; Sainsbury Wellcome Centre for Neural Circuits and Behaviour, University College London, London, UK

**Keywords:** blindness, geometry, shape perception, tactile perception

## Abstract

In a visual intruder task, regular quadrilaterals such as squares and rectangles are easier to process than matched shapes devoid of parallelism, symmetry or right-angles. This geometric regularity effect was found in various human groups, including preschoolers and uneducated adults, but not in non-human primates. It was proposed to reflect a fundamental ability to combine discrete geometric features into structured representations of geometric shapes using an abstract amodal language-of-thought (LoT) that also supports the acquisition of symbolic drawing and formal mathematics. Here, we tested a prediction of this hypothesis: blind participants should have the same intuitions of geometric regularity as sighted ones. To evaluate this prediction, congenitally blind and sighted (but blindfolded) adults underwent a tactile version of the visual quadrilateral intruder task. Among six tactile shapes, five of which were identical up to small size and rotation changes, participants were asked to identify a deviant shape defined by a fixed displacement of a single vertex, and to rate their confidence in their response. Both variables revealed a geometric regularity effect in both groups, and also correlated with previous results in the visual domain. Furthermore, a symbolic LoT model was a better predictor of tactile performance than a visual CNN model in blind participants. Thus, the geometric regularity effect develops in the absence of vision.

## INTRODUCTION

For centuries, philosophers such as Plato (428 b.C.–348 b.C.), Descartes (1596–1650) and Kant (1724–1804), have debated the origins of geometric knowledge in human cognition: are we born as genuine “geometric thinkers” or does this cognitive ability result from visual experience and education? In other words: are the basic geometrical notions of length, angle, parallelism, and symmetry inherent to our neural circuitry, or do we develop an understanding of these concepts through the process of education and visual experience? These important questions are still at the heart of an ongoing debate in contemporary experimental studies in cognitive neuroscience.

One source of data is the study of cognition following early sensory deprivation. The French philosopher Denis Diderot (1749) reported the example of a talented blind scientist and mathematician, Nicholas Saunderson (1682–1739), to argue for the abstract and sensory-independent nature of geometrical knowledge. In line with this insight, current experimental evidence suggests that intuitions about geometry can emerge in individuals without vision or formal education, and that may even be shared with non-human species (Amir et al., 2014; Dillon et al., 2013; Gibson et al., 2007; Izard & Spelke, 2009). These studies were, however, focused on a limited range of geometric properties and/or spatial navigation skills. For example, the seminal study of Landau et al. (1981) showed that a blind child could use spatial cues for orientation and navigation, but more recent investigations suggest that core geometrical knowledge can be divided into distinct systems for spatial navigation and for the perception of 2D shapes (Dillon et al., 2013; Spelke et al., 2010). In the latter domain, recent behavioral studies have investigated a larger number of Euclidean geometric properties using deviant-figure detection tasks (or intruder-task; Dehaene et al., 2006). For instance, all humans, regardless of education, could easily find the single display of non-parallel lines among parallel ones, and similarly performed the intruder task at a high-performance level for features such as length, right angle, curvature, etc. (Dehaene et al., 2006; see also Dillon et al., 2019; Izard et al., 2022). Using a tactile version of this intruder task, Heimler et al. (2021) reported that even congenitally blinds adults were able to discriminate different geometric features similarly to blindfolded subjects, such that performance was strongly correlated between the two groups. Similar results have been found also in blind children with an analogous tactile task (Marlair et al., 2021). In the same task, western preschool children (Izard & Spelke, 2009), as well as Amazonian populations with no access to formal geometric education such as the Munduruku also spontaneously differentiated many geometric concepts (Dehaene et al., 2006). Although western preschool children and the Munduruku performed less accurately than schooled adults, performance across trials was highly correlated among the three groups, suggesting a shared foundation of core geometry reasoning independent of formal education and age. This experimental evidence hence suggests that the role of vision and formal education are not of paramount importance for the development of basic aspects of the core geometric system, thus supporting the view that evolutionary ancient cognitive mechanisms may support our geometrical intuitions (e.g., Spelke et al., 2010).

Real-world objects, however, are rarely defined by a single feature (e.g., one angle or one axis of symmetry), but by the *combination* of multiple geometric relations—parallelism, orthogonality, angle equality, and so on—which together constrain their global shape. Perceptual systems can exploit these *compositional cues* to segment contours from clutter, to recognize objects despite partial occlusion or sensory noise, and to build robust, modality-independent representations. Beyond individual Euclidean geometric concepts, Sablé-Meyer et al. (2021) recently investigated how the *combination* of visual geometric cues such as parallelism, right angles and symmetry can influence the perception of regularity in geometrical shapes. These properties partially overlap with non-accidental visual features (Biederman, 1987; Lowe, 2012) which are image configurations that are invariant to changes in viewpoint and are therefore helpful to identify objects in the scene. Several authors have also investigated the concept of geometric shape regularity, from the origin of Gestalt psychology (e.g., Koffka, 1935; Metzger, 1941; see also Leeuwenberg, 1971) investigating how “*Pragnanz*” (or good-form) and the simplicity principle determine the perception of objects, to more recent quantitative works on how regularity and non-accidental properties affect object perception (Biederman, 1987; Feldman, 2003; Kubilius et al., 2014, 2017; Strother & Kubovy, 2012; Wagemans, 1992). Remarkably, it has been shown that humans spontaneously use regularities in the stimulus to compress information in working memory and achieve a smaller “minimal description length”, thus facilitating memorization, anticipation, and outlier detection (e.g., Al Roumi et al., 2023; Amalric et al., 2017; Feldman, 2000).

In line with those previous studies, Sablé-Meyer et al. (2021) reasoned that the perceived geometric regularity of a shape (e.g., a quadrilateral) would be directly related to the number of its geometrical non-accidental properties such as parallel lines, equal sides, equal angles, and right angles. To test their hypothesis, four different populations were assessed in a variant of the original intruder-task, using several quadrilaterals with different degrees of regularity. The results showed that children and human adults as well as populations with no formal western-like education such as the Himbas, were sensitive to geometric regularity in a visual intruder task. In all populations, regular shapes such as squares and rectangles (containing a large number of non-accidental properties) were identified faster and more precisely than irregular quadrilateral shapes (containing no non-accidental properties). This geometric regularity effect was absent in baboons, suggesting that only humans may represent shapes in a more abstract, symbolic form. Furthermore, baboon performance was well captured by a classic bottom-up convolutional neural-network (CNN) model of the ventral visual pathway, but an additional symbolic model (featuring the number of discrete geometric properties) was required to capture human performance (see also Campbell et al., 2024).

In line with this double dissociation, a two-stage model was proposed for geometric shape representations (Sablé-Meyer et al., 2021). The first stage, shared with animals and well-modeled by CNNs, would involve image recognition in ventral visual cortex. It would be followed, in humans only, by a second stage in which shapes are processed symbolically, as a mental program in a Language of Thought for geometry (LoT; Sablé-Meyer et al., 2022). This proposal is in line with previous notions of a “language of vision” (Cavanagh, 2021) or “grammar of perception” (Leeuwenberg, 1971). According to this view, mathematical and geometrical knowledge is ultimately represented amodally, in an internal symbolic language. Indeed, a growing number of studies suggest that basic geometric properties can help grouping and object segmentation, not only in the visual domain, but even in the tactile and auditory modalities (for a review see Gallace & Spence, 2011). In vision, for example, Gestalt principles such as good continuation and symmetry integrate local edges into surfaces; likewise, in haptics, sequential touch of individual edges becomes more informative when the brain “knows” how those edges are globally arranged in space (e.g., Gallace & Spence, 2011).

Yet not all the studies agree with the view that visual Gestalt principles also apply to the tactile modality (e.g., Cuijpers et al., 2003; Kappers, 1999) and the debate is still open because of the modest number of systematic studies focused on the sensitivity to grouping and non-accidental properties in tactile perception, compared to other modalities such as vision (Gallace & Spence, 2011). Although a clear sensitivity to some individual geometric features (e.g., symmetry, right angles, parallelism) has been found in blind children and adults using tactile intruder tasks (Heimler et al., 2021; Marlair et al., 2021), tactile memory tasks (Cattaneo et al., 2010), and other formal tactile batteries (Ballesteros et al., 2005; Mazella et al., 2016), it is still unknown whether the geometric regularity effect and the rules governing the *combination* of geometric features into a geometric shape can be found in other modalities than vision.

In this study, we tackled this issue by testing congenitally blind individuals (and blindfolded sighted participants as controls) in a tactile intruder-task similar to Sablé-Meyer et al.’s (2021) visual test and by deliberately manipulating combinations of properties so as to probe this integrative code. Specifically, we used a set of 3D-printed stimuli depicting 6 raised-line geometric shapes, one of which was a deviant shape. 11 different base shapes with a different degree of regularity were tested, with rectangles and squares containing the highest degree of regularity and irregular shapes containing the lowest degree of regularity. If sensitivity to geometric regularities emerge only from visual mechanisms, congenitally blind participants should show similar performance in identifying all the different shapes (e.g., deviants would be as hard to identify among squares than among irregular quadrilaterals). If, on the contrary, an amodal geometric language is involved, then the geometric regularity effect should be observed even when vision is absent, and the task is performed in the tactile modality.

Due to the difficulty of the task, we chose to provide participants with 25 seconds of free inspection time for each test stimulus. We measured the accuracy of perceptual decisions, and we also evaluated the participants’ subjective confidence (i.e., the estimation of the likelihood that their decisions would be correct; Yeung & Summerfield, 2012). The rationale is straightforward: participants should make fewer errors when selecting more regular shapes, and, correspondingly, their confidence should be higher when they choose regular shapes compared to more irregular ones. We also tested whether performance in the tactile modality was correlated with that in the visual modality in schooled adults, children (preschoolers and 1^st^ graders), adults without access to formal western education (Himbas), and baboons. If the geometric regularity effect is amodal, performance in the tactile intruder-task should be strongly correlated with that in the visual version of the same intruder-task. Finally, we correlated performance in the tactile task, for both blind and blindfolded groups, with the predictions of the two computational models used by Sablé-Meyer et al. (2021): a symbolic model and a CNN (CORnet_S, modeling the infero-temporal layer). We predicted that tactile performance in the blind should be primarily explained by the symbolic model, in line with previous work.

## METHODS

### Participants

A group of 9 congenitally blind participants (Mean Age = 46 years, SD = 12.6; 4 females, see Table 1) and 13 blindfolded sighted participants took part in the study (Mean Age = 27.3 years, SD = 3.95; 6 females). Blind participants were recruited from French associations for the blind. All participants were compensated for their participation in the study (15 euros/h) and were naïve about the purpose of the experiment. Each participant signed an informed consent document before the experiment began, and the study was conducted in accordance with the Declaration of Helsinki. The study was approved by the local ethical committee.

**Table 1.** Congenitally blinds sample description.

**Participant**	**Sex**	**Age**	**Cause of Blindness**	**Onset**	**Education**
*1*	m	30	Congenital Blindness/Genetic	birth	Bac+3
*2*	m	59	Leber Congenital Amaurosis	birth	Bac+5
*3*	f	52	Medical error/Incubator	birth	CAP
*4*	f	52	Aniridia	birth	Bac+3
*5*	m	56	Bilateral Retinoblastoma	birth	Bac
*6*	m	57	Toxoplasmosis	birth	Bac
*7*	m	50	Congenital Glaucoma	birth	Bac+2
*8*	f	37	Bilateral Microphthalmia	birth	Bac+3
*9*	f	21	Retinopathy of Prematurity	birth	Bac+2

Bac = final French examination before entering university. CAP = professional diploma after 11 years of education.

### Stimuli

**Figure 1.**
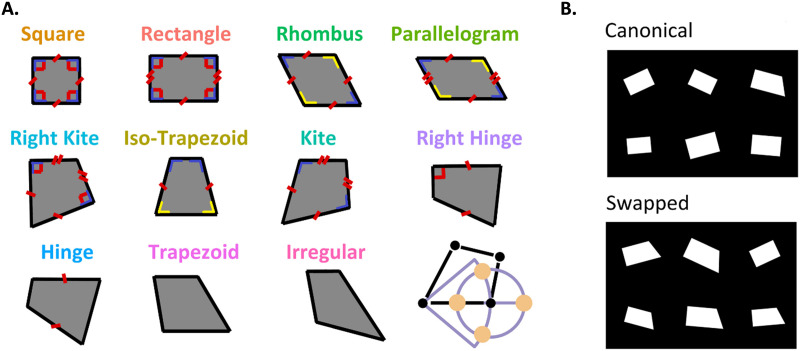
**(A) Basic geometrical shapes:** The original 11 shapes were generated as in Sablé-Meyer et al. (2021). The 11 quadrilaterals are ordered according to their number of geometrical regularities (parallelism, equal sides, equal angles, or right angles). For each quadrilateral, four deviants were generated by moving the bottom right corner by a fixed distance, thus shortening, lengthening, or rotating the bottom side. **(B) Type of Stimuli:** One-half of the stimuli were generated with a canonical disposition (five instances of one of the 11 reference base shapes, plus a single deviant) and the other half were swapped (five deviants, identical up to a rotation or scale change, plus a single reference shape).

**Figure 2.**
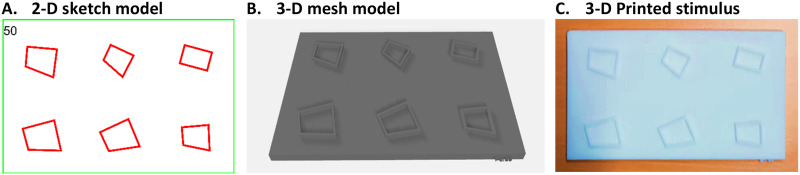
**Stimuli generation method: (A)** Original 2-D visual stimulus as generated in Sablé-Meyer et al. (2021). In this example, the deviant-shape is in the top-right corner. **(B)** 3-D visual mesh model conversion obtained adding a third dimension to the 2-D shapes. **(C)** Example of a printed stimulus card containing 6 raised-lines shapes. Stimulus size = 120 mm × 73.5 mm × 6 mm. Raised Line = 2 mm (height) × 1 mm (width).

The quadrilateral tactile stimuli were generated with the same custom Python script that drew the original 2-D stimuli from Sablé-Meyer et al. (2021). Visual displays were converted to 3-D models with Autodesk Meshmixer (3.4.35; Autodesk Inc., San Rafael, CA, USA) and professionally printed in-house with a 3-D printer (see Figure 1A and Figure 2). Each stimulus consisted of a solid plastic card (120 mm × 73.5 mm × 4 mm), with Velcro® strips applied in the back to fix the cards over the desktop to prevent displacement. On the top of each card, 6 shapes were printed with raised-lines of 2 mm height × 1 mm width. The experiment used a standardized set of 11 base quadrilateral shapes (Figure 1A; vertex coordinates of basic shapes are listed in Supplementary Materials in Sablé-Meyer et al., 2021). These shapes were matched for two key parameters. First, the average distance between all pairs of vertices (mean of six distances) was uniform across the 11 shapes, ensuring consistent overall size. Second, the length of the bottom edge was fixed across 9 of the 11 shapes, with exceptions made for the square and rhombus due to geometric constraints. The adopted constraints led to unique choices for certain shapes. Notably, the constraints did not strictly equalize other dimensions such as surface areas or perimeter. However, these features were found to be mostly uncorrelated with shape regularity (see *“**Nuisance variables analysis**”* section reported in Supplementary Materials for more details).

For each base shape, four deviant shapes were generated by modifying the position of the bottom-right vertex (Figure 1A). All deviant vertices were equidistant from the correct vertex location. Two deviant vertices altered the length of the bottom edge, while the other two changed their orientation while preserving the correct distance from the bottom-left vertex. The deviation distance was fixed for all deviant shapes and expressed as a proportion of the fixed average distance between all pairs of vertices (30%). In their default presentation, all shapes were centered on their center of mass, with the top edge being horizontal. Random variations were applied to introduce variability, including rotations [−25°, −15°, −5°, 5°, 15°, 25°] and scaling factors [0.875, 0.925, 0.975, 1.025, 1.075, 1.125]. We avoided 0° rotations to prevent reliance on the table edges or the 3D-printed plate borders, and we excluded larger rotation orientations to accommodate shapes with rotational symmetry (e.g., squares, who are unchanged under 90° rotation). The intruder itself was constrained to always use one of the 4 central values of rotation and scaling factors, in order to avoid extreme rotation angles or sizes which could be used as proxies to identify it. The position of the intruder was randomized (but not balanced) across the six spatial positions. One-half of the stimuli were generated with canonical disposition (five instances of one of the 11 reference base shapes, plus a single deviant) and the other half were swapped (five deviants, identical up to a rotation or scale change, plus a single reference shape; see Figure 1B) for a total of 88 different cards (11 shapes × 4 type of deviants × 2 canonical/swapped dispositions).

Overall, this stimulus design is based on our prediction that geometric regularity is a higher-order perceptual property—one not reducible to individual features but emerging from their structured interplay.

### Procedure

**Figure 3.**
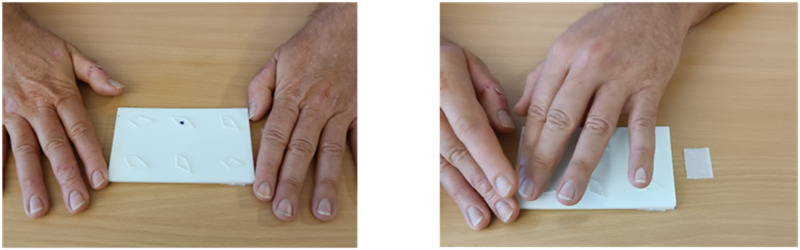
**Example of one experimental trial:** In the left panel, both hands were at the starting position. In the right panel, the participant explored the stimulus with both hands for 25 seconds.

Participants were comfortably seated near a desk in a quiet room. The instructions were read to each participant by the experimenter. Each trial involved touching a card containing six geometric figures. The task was to identify the figure with a different shape (intruder) among them. On each trial, a plastic card was fixed with Velcro® on the desk and placed along the participant's mid-sagittal line at a comfortable distance of 30 cm. Participants positioned their hands on both sides of the stimulus (starting position), and the trial began when the experimenter started the chronometer. The participants were thus allowed to explore the 6 shapes with both hands for 25 seconds (Figure 3). After this time, participants were asked to indicate the selected “intruder” with their finger, and subsequently they rated their confidence level on a Likert-scale, going from 1 (“I gave a random answer”) to 10 (“I’m totally sure”). Oral feedback was provided after each trial by the experimenter, revealing the correct intruder location in case of errors. After 3 initial training trials to familiarize the participant with the task, a total of 88 experimental stimuli were individually presented to participants in a pseudo-random order. The order of the cards was randomized off-line, separately for each participant, using a custom Python routine with the following constraints: (1) within each mini-block of 11 consecutive trials, one example of each of the 11 shapes was shown; (2) consecutive trials never showed the same shape. A total of 8 mini-blocks were presented. The whole experiment lasted around 55 minutes, with a short pause at the half of the experiment.

## RESULTS

### A Geometric Regularity Effect in the Tactile Modality (Blind vs Blindfolded)

**Figure 4.**
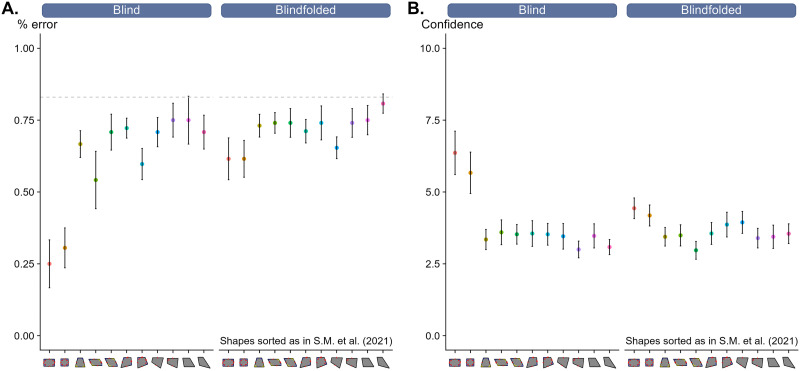
**(A)** Mean error rate in function of shape and group. Dashed gray line represents the chance level. **(B)** Confidence score in function of shape and group. Notice that the shapes under the x-axis were sorted as in the Experiment 2 reported in Sablé-Meyer et al. (2021). Bars represent ±1 standard error of the mean (SEM).

The data were analyzed using R-Studio (2015, v. 4.2.1; https://www.rstudio.com/) software package. For the ANOVA, when sphericity assumption was violated, we applied the Greenhouse-Geisser epsilon (ϵ) correction and we reported the original *F*, df and corrected *p*-values. Figure 4 shows the mean error rates for each shape and each group. Since we aimed at reproducing the visual-intruder task of Sablé-Meyer et al. (2021) in the tactile modality, the shapes were sorted according to the empirical regularity observed in the data of their Experiment 2. Data were submitted to a 11 × 2 mixed repeated-measures ANOVA with shape (square, rectangle, rhombus, parallelogram, right kite, iso-trapezoid, kite, right hinge, hinge, trapezoid, irregular) as within-subject variable and experimental group (blind vs. blindfolded) as between-subject variable. We found a significant main effect of group, *F*(1, 20) = 5.9, *p* = .025, *η*^2^_p_ = .22, as well as a significant main effect of shape, *F*(10, 200) = 9.45, ϵ = .57, *p* < .001, *η*^2^_p_ = .32,. The interaction between the two variables was also significant, *F*(10, 200) = 3.56, ϵ = .57, *p* = .003, *η*^2^_p_ = .15, Figure 4A.

To further investigate the relationship between performance and empirical complexity, we computed a subject-wise linear regression predicting error rate from complexity index. For each participant, the mean error rate was calculated at each level of empirical complexity, and a linear model was fit to estimate the slope of the relationship between complexity and error (one sample one-tailed *t*-test against zero). These slope estimates were then analyzed at the group level (Welch two-sample *t*-test).

As expected, error rates significantly increased with empirical complexity in both groups. In the blind group, the slope was strongly positive, indicating a robust complexity effect, *t*(8) = 6.12, *β* = 0.041, *p* < .001. A significant effect was also found in the blindfolded group, although to a lesser extent, *t*(12) = 2.37, *β* = 0.012, *p* = .018. An independent-samples Welch *t*-test confirmed that this complexity effect was significantly larger in blind participants compared to blindfolded ones, *t*(16.44) = 3.40, *p* = .0035. These results suggest that although both groups exhibited a sensitivity to complexity, blind participants were more affected, with performance deteriorating more steeply as empirical complexity increased.

Very similar results were found when we submitted the confidence ratings to the same 11 × 2 mixed repeated-measures ANOVA. The main effect of group was not significant, *F*(1, 20) = 0.19, *p* = .66, *η*^2^_p_ = .009, while the main effect of shape was significant, *F*(10, 200) = 17.26, ϵ = .40, *p* < .001, *η*^2^_p_ = .46. The interaction between the two variables was also significant, *F*(10, 200) = 5.44, ϵ = .40, *p* < .001, *η*^2^_p_ = .21, Figure 4B. As for the error rate, we conducted a subject-wise linear regression predicting confidence from empirical complexity. As predicted, confidence significantly decreased with empirical complexity in both groups. In the blind group, the slope was strongly negative, implying a strong complexity effect, *t*(8) = −4.43, *β* = −0.24, *p* = .0011. A significant effect was also found in the blindfolded group, although to a lesser extent, *t*(12) = −2.74, *β* = −0.052, *p* = .0088. As with error rate, an independent-samples Welch *t*-test revealed that the complexity effect on confidence scores was significantly greater in blind than in blindfolded participants, *t*(9.96) = −3.27, *p* = .0083. Finally, a multiple linear regression with experimental group and error rate as predictors of confidence scores revealed that higher errors rate significantly predicted a decrease in confidence, *t*(18) = −9.97*, β* = −5.8, *p* < .001, Figure S1. Here, the group effect and the interaction were not statistically significant (*p* > .05), suggesting that the relationship between error rate and confidence was similar in both groups.

Further analyses confirming the main results of this section are reported in the Supplementary Materials.

### Correlation Between Tactile and Visual Performance in Blind Group

We tested whether the geometric regularity pattern obtained in the tactile modality was similar to the pattern found in the visual modality. Data of the visual intruder-detection task for French adults, preschoolers & 1^st^ graders, Himbas and baboons were collected by Sablé-Meyer et al. (2021) and are available on-line.

**Figure 5.**
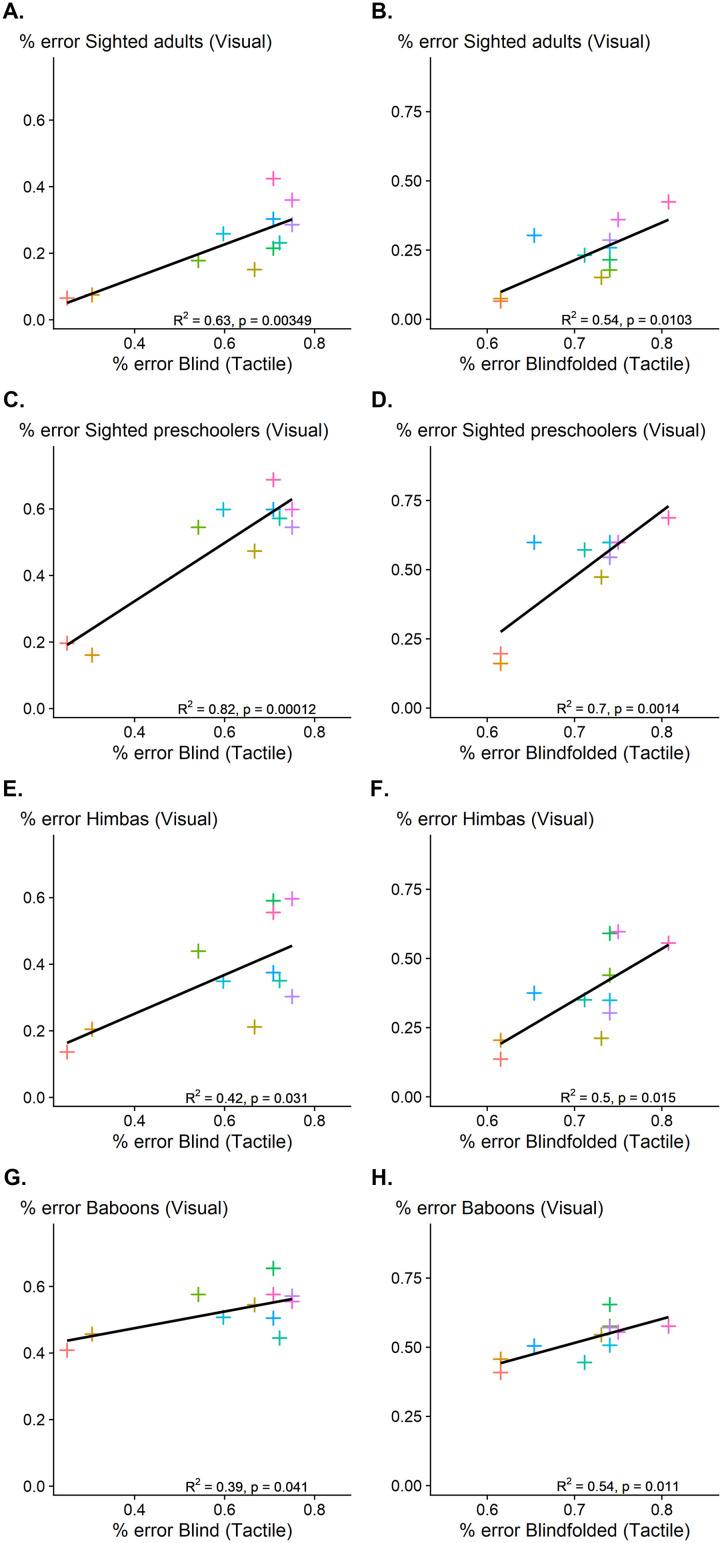
Correlation between the mean error rate in the tactile modality and the mean error rate in visual modality across groups (sighted adults, preschoolers, Himbas and baboons) for both blind **(Left column)** and blindfolded participants **(Right column)**. The black line represents the line of best fit.

We first correlated the error rate in the tactile task of the blind group over the 11 shapes with the error rate of sighted adults over the 11 shapes in the visual-task (Sablé-Meyer et al., 2021, Experiment 2). The two were highly correlated (*R*^2^ = .54, df = 9, *p* = .0103, Figure 5A), suggesting that the same shapes that were harder to identify in the tactile modality were also harder to identify in the visual modality.

We next tested whether a similar association was present independently of language knowledge and age. Performance in the tactile modality of blind adults strongly correlated with that in the visual modality for preschoolers (*R*^2^ = .70, df = 9, *p* = .0014, Figure 5C) and 1^st^ graders (*R*^2^ = .72, df = 9, *p* = .0008, Figure S2A) and was modestly correlated with the Himbas’ (*R*^2^ = .49, df = 9, *p* = .015, Figure 5E).

Finally, we tested whether the cross-modal correlation was also presented across species. We found that the performance of baboons in visual modality was significantly correlated with the performance of blind participants in the tactile modality, (*R*^2^ = .54, df = 9, *p* = .011), although the slope was very shallow (Figure 5G).

### Correlation Between Tactile and Visual Performance in Blindfolded Group

Similar to the previous analysis, we found that tactile performance in the blindfolded significantly correlated with the visual performance of sighted adults, (*R*^2^ = .63, df = 9, *p* = .003), preschoolers, (*R*^2^ = .82, df = 9, *p* < .001), 1^st^ graders, (*R*^2^ = .84, df = 9, *p* < .001), and the Himbas (*R*^2^ = .42, df = 9, *p* = .03; see Figure 5 right column and Figure S2B). A modest but still significant correlation was also found with baboons’ performance, (*R*^2^ = .39, df = 9, *p* = .04, Figure 5H). Thus, the same shapes that were harder to identify in the tactile intruder task for the blindfolded were also harder to identify for all the sighted groups in the visual modality.

### Computational Modeling of Tactile Performance

In previous work with the visual geometric intruder task, we observed that, in humans, the variations in performance across shapes were well predicted by a symbolic model of geometric regularities, whereas in baboon, systematic variations also existed but were uncorrelated with geometric regularities and well predicted by a CNN. Here, we replicated this analysis on the present data. The hypothesis of an amodal geometric language common to all humans predicted that the geometric model should predict the tactile performance of blind participants better than the CNN.

We start by briefly summarizing the two models. The CNN assumes that quadrilaterals are processed by standard image recognition mechanisms in the ventral visual pathway, while the symbolic model assumes an additional level of discrete, symbolic processing of nonaccidental geometrical properties. We modeled the ventral visual pathway using CORnet, a top-scoring CNN on Brain-Score.org. This model, pretrained on ImageNet, was used to simulate the visual outlier task. Without retraining, we input the same six images shown to participants and collected activation vectors from each CNN layer. The image whose vector deviated most from the mean of the other 5 images was identified as the intruder, yielding a predicted error rate per shape and model layer. The symbolic model, on the other hand, was based on categorical perception of geometric properties (e.g., parallelism, right angles, etc.). Each quadrilateral was encoded as a symbolic vector of geometric features (i.e., 0s and 1s for the absence or presence of equal angles, equal sides, parallelism, and right angles). The ease of the intruder detection task was then predicted by the L1 distance (Manhattan distance) between the symbolic vectors coding for the reference and deviant shapes (for more details, see Sablé-Meyer et al., 2021).

**Figure 6.**
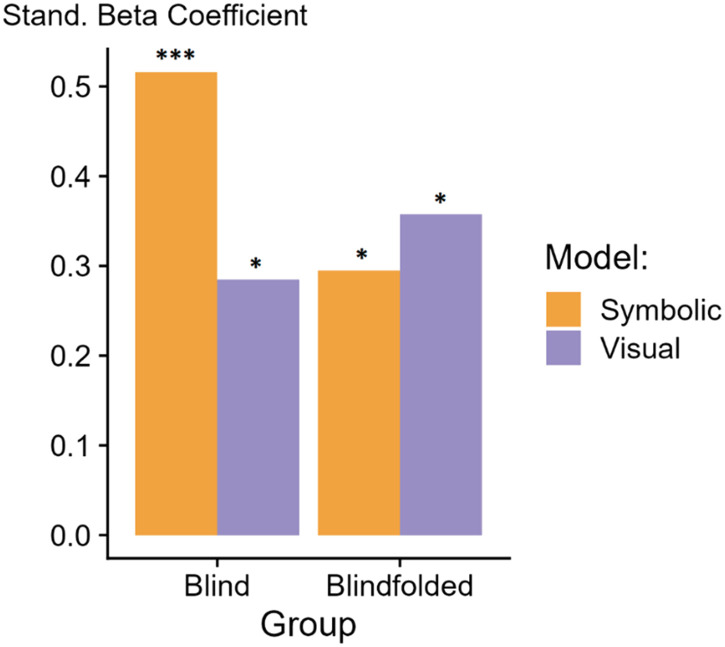
Standardized regression weights (β) obtained with multiple regression fitted over the data in the tactile-task from the blind and the blindfolded groups across 44 data points (11 shapes × 4 outlier types), using the symbolic and CNN models as predictors. Stars indicate the significance level. *p < .05; **p < .01; ***p < .001; *ns* = non-significant.

As in previous work, we ran a multiple linear regression analysis across 44 data points (11 shapes × 4 outlier types) with the performance achieved by the models (symbolic or CNN) as predictors and the performance in the tactile modality as dependent variable for each group (Blind and Sighted control group). Standardized beta coefficients were computed with the *QuantPsyc* package in R (Fletcher & Fletcher, 2010). As can be observed in Figure 6, the standardized beta coefficient obtained for the performance of blind participants was statistically significant for the CNN model, *t*(41) = 2.3*, β_standardized_* = 0.28, *p* = .026, but was substantially larger for the symbolic model, *t*(41) = 4.1*, β_standardized_* = 0.51, *p* < .001. A post-hoc one-tailed z-test revealed a marginal significance difference between the standardized predictors, *z* = 1.3, *p* = .09. This indicates that while the CNN model provides some predictive power, the symbolic model is a slightly better predictor of tactile performance in blind participants.

For blindfolded participants, the standardized beta coefficient was slightly higher for the CNN model, *t*(41) = 2.54, *β*_*standardized*_ = 0.35, *p* = .014, than for the symbolic model, *t*(41) = 2.1, *β*_*standardized*_ = 0.29, *p* = .041, although a post-hoc one tailed z-test revealed no significant difference between the standardized predictors, *z* = −0.31, *p* = .37.

Overall, this pattern of results suggests that blind participants, who are more experienced in recognizing shapes by touch, rely more on abstract geometric processing, making the symbolic model a better predictor of their performance in the tactile task. Conversely, sighted participants, using touch alone, may struggle to access integrate geometric features and instead rely more on visual imagery, thus explaining that their tactile performance is slightly better predicted by the CNN model.

## DISCUSSION

In this work, we investigated whether the geometric properties of shape stimuli are encoded independently of input modality. We found that the same combinations of geometrical cues affecting the *visual* perception of geometric regularity (Sablé-Meyer et al., 2021) are also exploited in the *tactile* modality. First, a geometric regularity effect affected performance in the tactile intruder task in both congenitally blinds and blindfolded sighted participants. This finding rules out a causal role of vision in the development of the geometric regularity effect, and strongly suggests that the perception of shape regularity does not stem exclusively from visual experience of the world. Rather, the perception of shape regularity is driven by the Gestalt principle of good-form or *Pragnanz* even in the tactile modality (Gallace & Spence, 2011). Although a few examples of tactile shape recognition are provided by Metzger (2006), to our knowledge, no empirical study has ever extensively addressed this topic so far in the tactile modality.

Interestingly, the performance of the blind group was overall better than the sighted controls, suggesting that compensatory cognitive mechanisms or brain plasticity due to long-term blindness and enhanced tactile stimulation may boost the performance of the blind subjects in the tactile task (e.g., Goldreich & Kanics, 2003; Norman & Bartholomew, 2011). Accordingly, the performance in the tactile modality of blind participants was better predicted by our proposed symbolic model, while the CNN and symbolic models were on a par in predicting the performance of blindfolded participants. This finding suggests that, while blind participants, being experienced with touch, readily exploit geometric features such as symmetry and parallelism and their combinations in the tactile modality, sighted participants find it harder and remain influenced by the visual images of shapes.

Additionally, we found that performance in the tactile modality was correlated with performance in visual modality across all the populations examined. That is, irregular shapes were hardly identified in both visual and tactile modality while regular shapes were easily identified in both modalities, independently of the population examined, similar to previous studies focused on general geometric concepts (Heimler et al., 2021). This finding fits with previous visual-tactile object recognition experiments documenting shared brain mechanisms subtending both visual and tactile geometrical shape-information processing (Amedi et al., 2010; James et al., 2007; Lacey et al., 2009).

In both cases, the strength of the correlation was greatest when the performance of children in the visual modality was considered as a predicted variable. This was mostly, but not solely, driven by better performance with squares and rectangles, suggesting that those shapes are early and fundamental building blocks of geometry that are easiest to perceive in both children and blind subjects. A more mature visual system may enhance geometry perception by allowing for the parallel processing of the global shape of an object and turning it into an abstract mental program. Several other cognitive factors such as reading and letter identification (which strongly taps into non-accidental properties; Dehaene, 2009; Szwed et al., 2009) may have boosted this ability in the visual modality compared to the tactile modality.

Performance in the tactile modality was also slightly but significantly correlated with the performance of baboons in the visual modality for both congenitally blinds and sighted blindfolded participants. This small effect (Figure 5G) fits with the idea that a mixture of two distinct strategies (perceptual and geometry-driven) can be used to solve the task. In our previous work (Sablé-Meyer et al., 2021), we similarly found that, in the less educated participants (children and Himba), the geometry-driven strategy was less dominant that in participants with advanced education. It is likely that performance is slightly modulated in a similar way in baboons and humans due to the sharing of evolutionarily ancient abilities such as non-accidental geometrical properties detection. This would be in line with previous studies (Amir et al., 2011; Biederman, 1987; Kayaert et al., 2005; Kim & Biederman, 2012), where lateral-occipital (in humans) or infero-temporal (in monkeys) cortex exhibited a sensitivity to nonaccidental changes. However, only humans presented a strong geometric regularity effect in the visual modality, suggesting that baboons may lack the ability to combine multiple geometric cues into a higher-level mental program, hence leading to no geometric regularity effect.

Although all correlations between tactile performance and previously published visual data were significantly positive, their magnitudes also varied across groups—from preschoolers (*R*^2^ ≈ .70–.84) to blind versus blindfolded adults (*R*^2^ ≈ .42–.49) to non-human primates (*R*^2^ ≈ .39–.54) (see Figure 5). We believe this variability reflects several interacting factors. First, it is likely that, in some blind individuals at least, geometric knowledge remains at an early stage of development. This would explain the greater similarity between sighted preschoolers and blind adults. By adulthood, individuals bring varied educational backgrounds, imagery habits, and task-specific heuristics that can introduce a change into their geometric knowledge and slightly weaken their correspondence between visual and tactile tests. Likewise, congenitally blind participants, highly practiced in haptic shape analysis, show more consistent, geometry-focused exploration than sighted blindfolded participants, who are inexperienced with the tactile modality and thus exhibit less reproducible results.

In spite of these variations in correlation strength, however, the most important finding for the present study as well our past cross-cultural comparisons is that a core sensitivity to geometric regularity is robustly found across modalities and even in the absence of exposure to teaching of geometric regularity. This uniformly positive pattern across five very different populations, despite the many sources of variation, underscores the presence of an amodal geometric core and echoes recent critiques of cultural-byproduct claims that overly rely on limited or inconsistent cross-cultural data (Amir & Firestone, 2025).

Intriguingly, whereas sighted-to-sighted correlations spanned the full geometric regularity gradient, blind-to-sighted correlations were driven primarily by the most regular and most irregular shapes. We believe this pattern reflects the extensive tactile experience with these forms of our blind participants—experience that our blindfolded sighted participants lacked. As expected, performance peaked for the most regular shapes (squares and rectangles), which aligns with our hypothesis. The pronounced “step” in performance for these highly symmetric and right-angled forms, in line with our theory, likely arises from the superior memorization of those highly regular and salient shapes by blind individuals, amplified by their specialized exploration and encoding strategies in the tactile modality (see also Szubielska & Zabielska-Mendyk, 2018). Similarly, these right-angled shapes (e.g., squares, rectangles) are more easily discriminated by sighted children due to their geometric salience and their early emergence as core categorical representations in cognitive development. In adulthood, the performance gap between squares or rectangles and other shapes tends to diminish, likely due to the development of more abstract and flexible geometric representations that facilitate the mental representation of shapes of intermediate geometric regularity (e.g., trapezoid, parallelogram, etc).

Many empirical studies have highlighted the importance of specific non-accidental geometric properties such as parallelism, collinearity, right angles, and symmetry in visual or tactile perception (Amir et al., 2012; Feldman, 2007; Heimler et al., 2021; Kubilius, Sleurs, & Wagemans, 2017; Machilsen et al., 2009). However, those previous studies primarily investigated only isolated features. For example, Kubilius et al. (2017) focused on the type of junction (e.g., X, T, or L-shaped) in the visual modality while Heimler et al. (2021), investigated single geometric features such as symmetry, right angles, parallelism and shape in the tactile modality. Hence, there is a lack of knowledge about the rules governing the integration of multiple non-accidental cues. Our study suggests that these non-accidental properties are recombined in the tactile modality according to the same rules or “grammar of perception” found in visual modality. Indeed, the symbolic model proposed by Sablé-Meyer et al. (2021) predicts that perceived regularity is linked to the total number of individual non-accidental features in a shape. This is reminiscent of what has been observed in Gestalt psychology when multiple grouping cues are present in the visual stimulus: such cues may add their individual effects, leading to an overall stronger effect which is roughly the sum of the single effects (e.g., Adriano & Ciccione, 2024; Kubovy & van den Berg, 2008; Luna & Montoro, 2011; Quinlan & Wilton, 1998; Rashal et al., 2017; Schmidt & Schmidt, 2013). Similar interactions between grouping cues have also been observed in the tactile modality (Prieto et al., 2019). Furthermore, Biederman (1987) showed that performance in recognition of degraded objects is related to the number of components presented in the objects. When the number of components was low, performance dropped in proportion. This suggests that the overall number of components or non-accidental properties in the object should influence its recognition, as we show even for the perception of simple quadrilaterals in the tactile modality. Additionally, prior research (Leeuwenberg & van der Helm, 2013; Leeuwenberg et al., 1994) suggests that these regular object configurations can be efficiently encoded in working memory using more concise representations, which require fewer parameters.

Various computational factors that affect perceptual grouping in object recognition tend to converge through mechanisms that either reduce complexity or enhance regularity. Several researchers, including Hochberg and McAlister (1953), Leeuwenberg (1971), and Hatfield and Epstein (1985), have argued that perceptual grouping helps to minimize complexity or maximize regularity (see also Feldman, 1997, 2009).

Over and above the different theoretical approaches to object regularity, our findings provide new insights into the principles that govern perceptual grouping and object perception in the tactile modality. Combinations of geometric properties play a role in grouping and object perception even when the object is sensed with the fingers, similarly to what has been found in the visual modality. In turn, these combinations trigger the geometric regularity effect that we have found. Hence, we hypothesize that the geometric regularity effect rests on an *amodal* mechanism that can emerge even without vision. The present results suggest that feature repetition, concatenation, and recursion, which are thought to lie at the heart of the proposed grammar (Sablé-Meyer et al., 2021; see Hafri et al., 2023 for an overview of compositionality in vision), may govern shape regularity in both visual and tactile modalities, a proposal that should be evaluated in further studies. It would also be interesting to conduct neuroimaging studies with the same visual and tactile stimuli, as our hypothesis would predict that the same brain areas would be involved in this amodal geometry system (for a first set of MEG and fMRI studies with visual stimuli, see Sablé-Meyer et al., 2024).

We close by noting that we only explored here a specific set of geometric shapes, quadrilaterals, for which a description in terms of geometric features and their composition is most appropriate. However, several other models have been proposed for the mental representation of shape in a broader sense, from a shape’s contour (e.g., Kourtzi & Kanwisher, 2001) to its medial-axis skeleton (Ayzenberg et al., 2019; Blum, 1973; Feldman & Singh, 2006) or its purely topological properties (Yousif & Brannon, 2025). By design, the present shapes all shared similar contour, skeletal and topological properties, thus allowing us to focus on their geometric properties, and implying that these other models would not easily explain the present results. However, it would be interesting to extend the present approach and ask whether, for instance, congenitally blind also rely on the shape skeleton during the perception of a broader range of shapes.

## CONCLUSIONS

This study aimed to test the hypothesis that the human cognitive system uses perceptual regularities to encode shape stimuli independently of the input modality. In agreement with this proposal, we found that congenitally blind and blindfolded sighted participants exhibit similar geometric regularity patterns in an outlier task in the tactile modality. We also observed a strong correlation between performance in the tactile and visual modalities across different populations. The same symbolic geometry model predicted performance in both cases. In sum, irregular shapes were difficult to identify, while geometrically regular shapes were easily recognized in both tactile and visual modality. Our findings suggest that high-level geometric primitives are used across visual and tactile modalities, independently of visual experience, highlighting a shared *amodal* cognitive mechanism for the encoding of geometric features.

## Supplementary Material


