# Supplementary material for: Sensitivity to Geometric Shape Regularity Emerges Independently of Vision
Source: Open Mind (Camb). 2025 Oct 17;9:1711–27. doi: 10.1162/OPMI.a.39 (PMC12618013; doi:10.1162/OPMI.a.39)
Supplement: Supplementary file 1 [file opmi-09-1711-s001.pdf]

Supplementary Materials

Sensitivity to geometric shape regularity emerges independently of vision.

Andrea Adriano[1], Mathias Sablé-Meyer[1, 2, 3], Lorenzo Ciccione[1, 2], Minye Zhan[1] & Stanislas Dehaene[1, 2*]

[1] Cognitive Neuroimaging Unit, CEA, INSERM, Université Paris-Saclay, NeuroSpin center, 91191 Gif/Yvette, France

[2] Collège de France, Université Paris Sciences Lettres (PSL), 75005 Paris, France

[3] Sainsbury Wellcome Centre for Neural Circuits and Behaviour, University College London, London, UK

1. Supplementary figures from "Results" section.

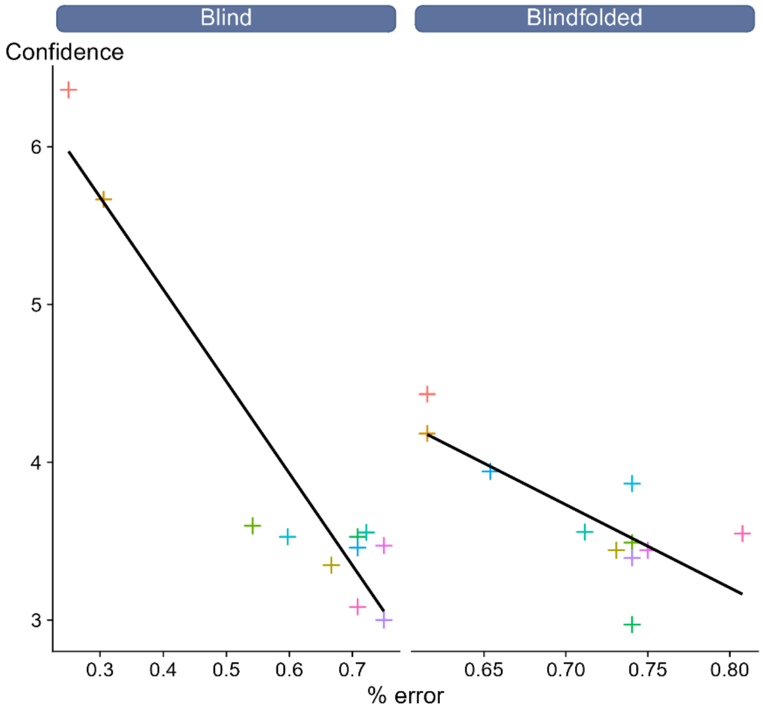

Figure S1: Correlation between mean error rate and confidence in the tactile intruder task in function of the group. The black line represents the line of best fit.

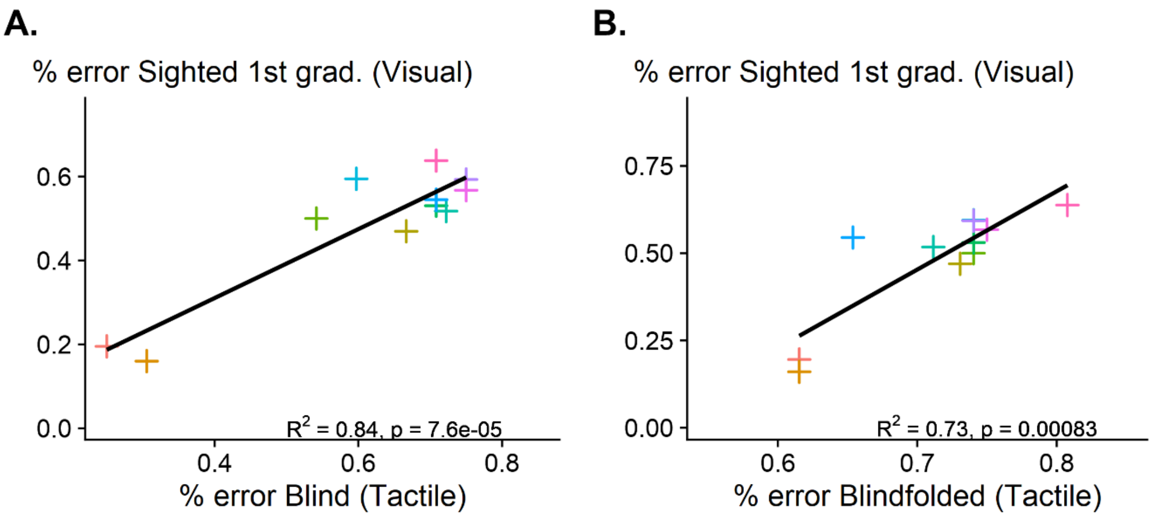

Figure S2: A) Correlation between the mean error rate in the tactile modality (blindfolded) and the mean error rate in visual modality (1st graders). **B)** Correlation between the mean error rate in the tactile modality (blind) and the mean error rate in visual modality (1st graders). The black line represents the line of best fit.

2. Supplementary Analysis.

We conducted both a leave-one-out analysis and a Pearson correlation across participants in each group, as well as the average split-half correlation (i.e., the mean across all possible split-half combinations). As expected, the effect of regularity was very strong and highly consistent among the blind participants—who are extensively trained in shape recognition via touch. The leave-one-out analysis showed that, regardless of which participant was excluded, the effect remained significant, indicating that the effect is robust despite our small sample size (Table S1). The Average split half reliability was 0.75. Moreover, the heat map of pairwise correlations confirms a uniformly strong relationship across all subject pairs (Figure S3). The effect also appeared in the blindfolded group, albeit to a lesser degree, consistent with our theoretical predictions. In this group, Participant 13 exerts the greatest influence: omitting them causes the p-value to approach marginal significance (Table S2). The Average split half reliability was 0.73. Furthermore, the heat map of pairwise correlations reveals weaker inter-subject agreement among the sighted participants, consistent with the idea that they are less proficient at discriminating shapes by touch (Figure S4). Finally, a *t*-test between the mean of the regression slope after the leave-one-out procedure, revealed a significant difference in the slope between the 2 groups, $t(12.2) = 30.54$, $p < .001$ (Figure S5).

Subject Left Out	Intercept	Slope	P value	Adj. R^2
14	0.3252841	0.04474432	**0.005431536**	0.5500187
15	0.3494318	0.04048295	**0.007280272**	0.5215119
16	0.3340909	0.04446023	**0.004374077**	0.5700807
17	0.3823864	0.03948864	**0.006432075**	0.5337606
18	0.3846591	0.03721591	**0.007657916**	0.5164281
19	0.3750000	0.03977273	**0.003663440**	0.5858893
20	0.3482955	0.04375000	**0.006177528**	0.5376913
21	0.3752841	0.04090909	**0.002604892**	0.6147868
22	0.3846591	0.04076705	**0.006366023**	0.5347683

Table S1: Leave-one-out regression analysis for the Blind group.

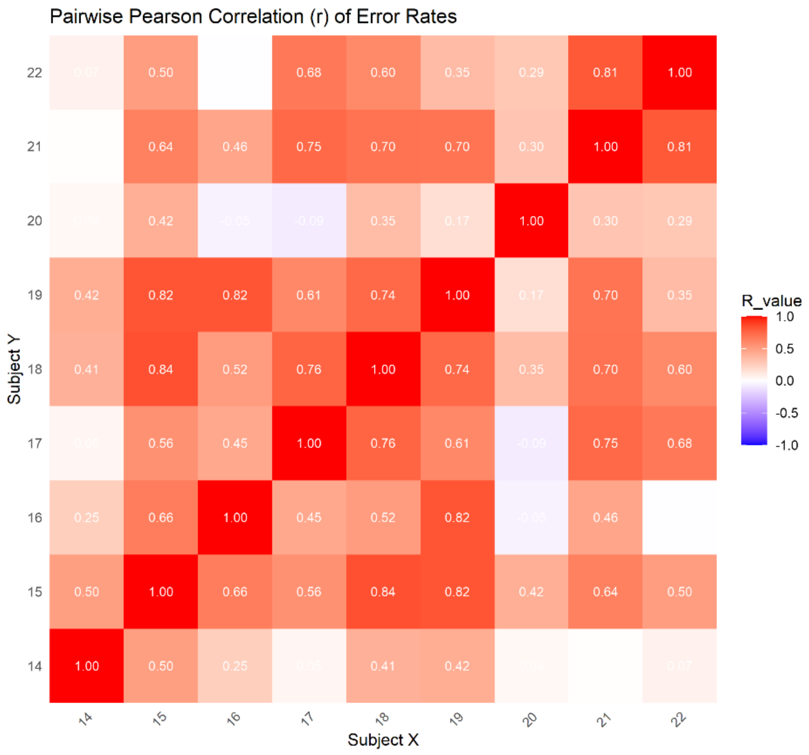

Figure S3: Inter-Subjects correlation analysis for the Blind group.

Subject Left Out	Intercept	Slope	P value	Adj. R^2
1	0.6558712	0.009375000	**0.03126020**	0.3546716
2	0.6253788	0.013352273	**0.03205666**	0.3514098
3	0.6363636	0.011837121	**0.02362859**	0.3900757
4	0.6373106	0.012310606	**0.02295011**	0.3936679
5	0.6357955	0.013352273	**0.02495516**	0.3832935
6	0.6320076	0.013352273	**0.03643475**	0.3346112
7	0.6321970	0.014109848	**0.01251995**	0.4644320
8	0.6357955	0.011931818	**0.02391103**	0.3886056
9	0.6285985	0.012973485	**0.02499991**	0.3830700
10	0.6607955	0.010606061	**0.02209729**	0.3983106
11	0.6356061	0.012594697	**0.01463289**	0.4469367
12	0.6214015	0.014488636	**0.01335006**	0.4572892
13	0.6742424	0.009943182	**0.06184645**	0.2616826

Table S2: Leave-one-out regression analysis for the Blindfolded group.

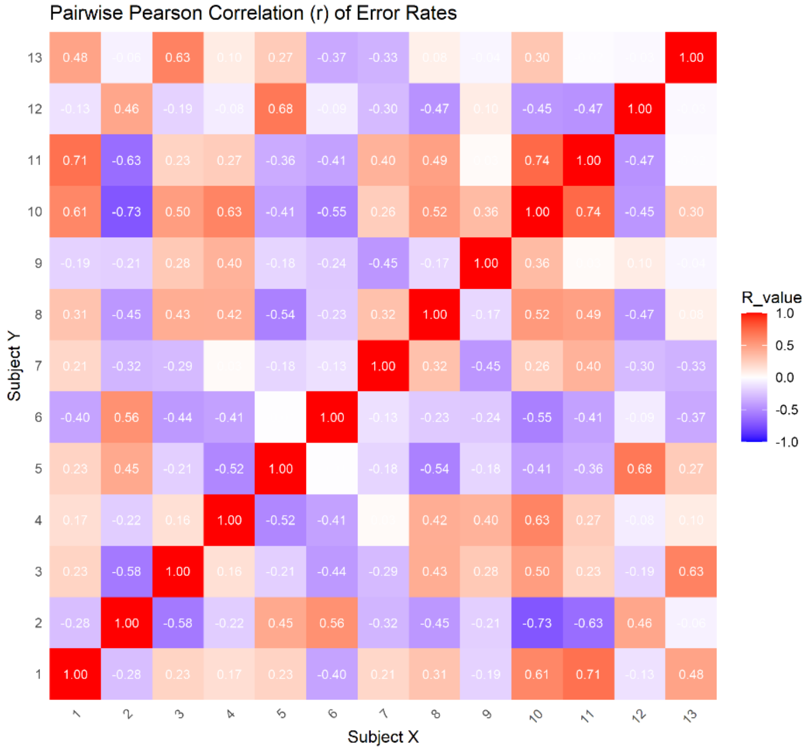

Figure S4: Inter-Subjects correlation analysis for the Blindfolded group.

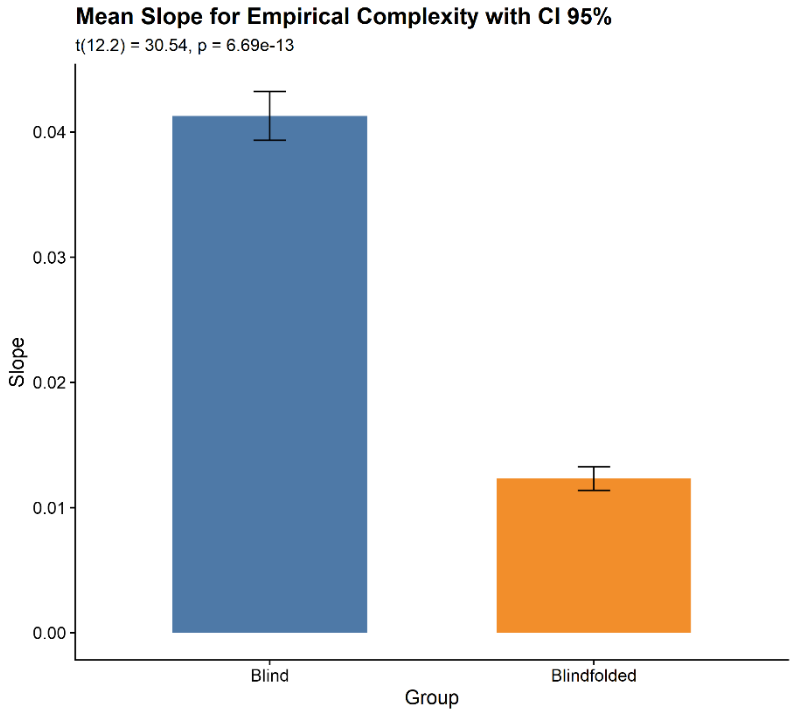

Figure S5: Mean of the regression slope after the leave-one-out procedure in function of the group. The error bars represent the 95% confidence interval.

3. Nuisance variables analysis.

As a control, we tested the impact of experimental nuisance variables such as rotation angle, the scaling factor, the position of the intruder shape in the card, and the canonical/swapped pattern. Since we did not have strong predictions about differences between groups in these experimental factors, we collapsed the between-groups variable. First, we run an 11 × 2 repeated-measures ANOVA with the shape and the type of display (canonical or swapped) as independent variables and error rate as dependent variable. We found a significant effect of the shape, $F(10, 210) = 7.1$, $\varepsilon = 0.49$, $p < .001$, $\eta^2_p = .25$, Figure S6A, but no main effect of display type, nor any interaction (all $p > .05$). There was a strong correlation between performance with the canonical display and the swapped display, $F(1, 9) = 15.6$, $R^2 = .63$, $\beta = 0.84$, $p = .0033$, Figure S6B.

Two other one-way repeated measures ANOVAs were performed with the rotation angle (-15, -5, 5, 15) or the intruder position (0 = top left, 1 = top middle, 2 = top right, 3 = bottom left, 4 = bottom middle, 5 = bottom right) as within factor respectively and the mean error rate as dependent variable. We found that neither the rotation angle, $F(3, 63) = 2.24$, $p = .09$, $\eta^2_p = .09$, Figure S7A, nor the position, $F(5, 105) = 2.03$, $p = .08$, $\eta^2_p = .088$, Figure S7B, affected the performance. We also checked whether the scaling factor affected the overall performance. A linear regression analysis showed no relation between the scaling factor and the mean error, $F(1, 2) = 1.05$, $R^2 = .34$, $\beta = 0.38$, $p = .41$. Finally, we also tested whether mean individual perimeter and area of the shapes (taking into account the actual scaling factor applied to the edge length by the random permutation) could explain the geometric regularity effect. Results of the linear regression show no relationship between the mean error rate and either the mean actual perimeter, $F(1, 9) = 2.9$, $R^2 = .24$, $\beta = 0.49$, $p = .12$, Figure S7C, or the mean actual area of the target shapes, $F(1, 9) = 0.96$, $R^2 = .09$, $\beta = -0.25$, $p = .35$, Figure S7D. Overall, we found that experimental nuisance factors had a minimal impact over the performance in the intruder detection task.

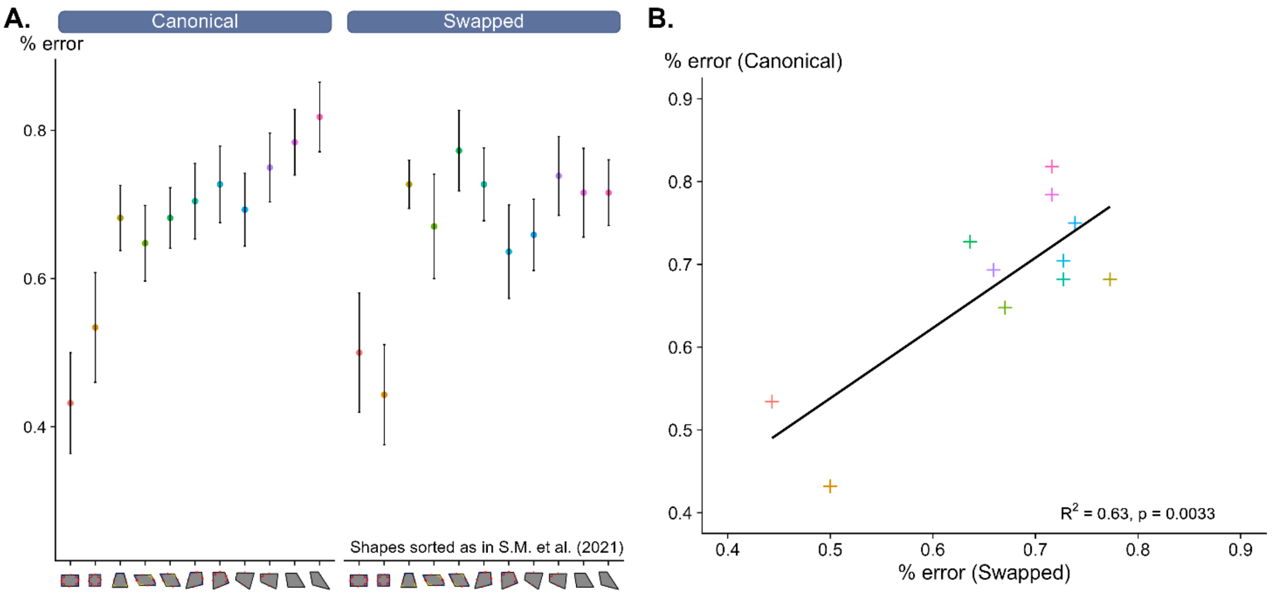

Figure S6: A) Mean error rate in function of the shape and the type of display. Bars represent ±1 SEM. **B)** Correlation between mean error rate in the swapped and the mean error rate in canonical display. The black line represents the line of best fit.

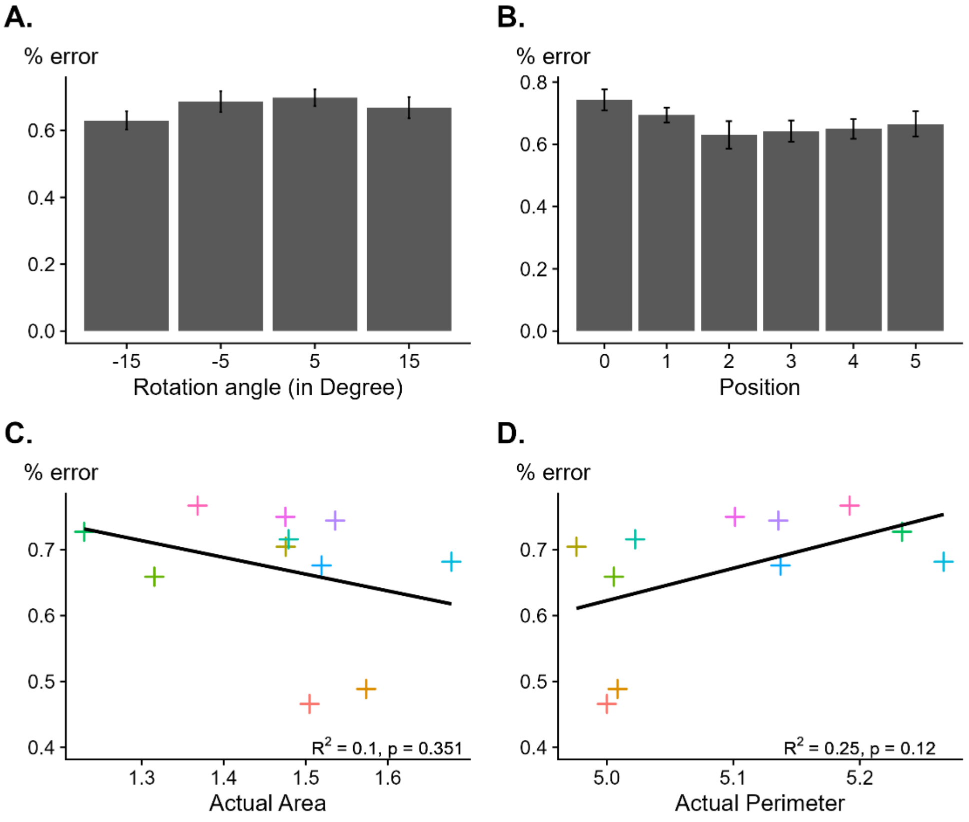

Figure S7: A) Mean error rate in function of intruder rotation angle (in degree). **B)** Mean error rate as a function of intruder position (0 = top left, 1 = top middle, 2 = top right, 3 = bottom left, 4 = bottom middle, 5 = bottom right). Bars represent ±1 SEM. **C)** Correlation between mean error rate and the actual perimeter. **D)** Correlation between mean error rate and the actual area. The black line represents the line of best fit.
